# Friedelin in *Maytenus ilicifolia* Is Produced by Friedelin Synthase Isoforms

**DOI:** 10.3390/molecules23030700

**Published:** 2018-03-20

**Authors:** Thaís B. Alves, Tatiana M. Souza-Moreira, Sandro R. Valentini, Cleslei F. Zanelli, Maysa Furlan

**Affiliations:** 1 Instituto de Química, Univ. Estadual Paulista-UNESP, Rua Prof. Francisco Degni, 55, Quitandinha, Araraquara, SP 14800-060, Brazil; thais.barboni@gmail.com (T.B.A.); mayfurlan@gmail.com (M.F.); 2 Faculdade de Ciências Farmacêuticas, Univ. Estadual Paulista-UNESP, Rod. Araraquara-Jaú km 1, Araraquara, SP 14801-903, Brazil; souzatm@gmail.com (T.M.S.-M.); valentsr@fcfar.unesp.br (S.R.V.)

**Keywords:** polymorphism, triterpene, *Maytenus ilicifolia*, *Saccharomyces cereivisiae*, heterologous expression, oxidosqualene cyclase

## Abstract

Triterpenes are interesting compounds because they play an important role in cell homeostasis and a wide variety exhibiting defense functions is produced by plant secondary metabolism. Those same plant secondary metabolites also exhibit biological properties with promising therapeutic potential as anti-inflammatory and antitumor agents. Friedelin is a triterpene ketone with anti-inflammatory and gastroprotective activities and it is a precursor of relevant antitumor quinonemethides. Although many triterpene synthases have been described, only two friedelin synthases were characterized and there is no information about their genomic features and alleles. In the present work, we aimed to identify the gene and new isoforms of friedelin synthase in *Maytenus ilicifolia* leaves to be functionally characterized in *Saccharomyces cerevisiae*. The gene sequence analysis elucidated the exon/intron structure and confirmed the presence of single nucleotide polymorphisms with four non-synonymous mutations outside the active site of the enzyme. Therefore, two new isoforms were observed and the heterologous production of the enzymes in yeast showed similar production of friedelin. This first description of different alleles of the gene of friedelin synthase in *M. ilicifolia* can guide their validation as markers for friedelin-producer specimens.

## 1. Introduction

**Figure 1:**
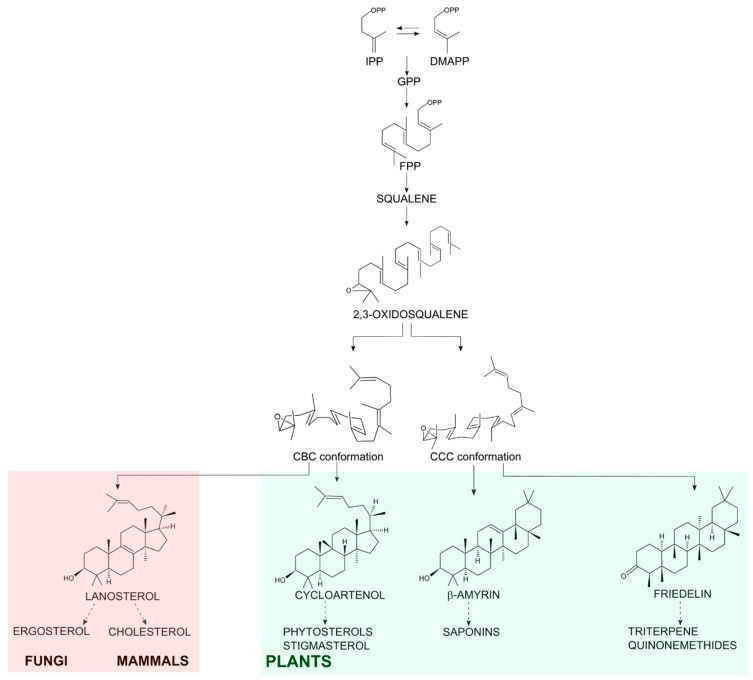
Schematic biosynthesis of steroidal and pentacyclic triterpene. Conjugation of isopentenyl pyrophosphate (IPP) with its isomer dimethylallyl pyrophosphate (DMAPP) generates the 10-carbon molecule geranyl pyrophosphate (GPP). GPP conjugation to another IPP monomer generates the C-15 farnesyl pyrophosphate (FPP). Then, the conjugation of 2 FPP molecules generates the C-30 precursor squalene. After oxidation of squalene, 2,3-oxidosqualene is cyclized by the OSCs in the organism. In fungi, mammals and plants the chair-boat-chair (CBC) conformation results in the steroidal lanosterol (in the light red shadow) or cycloartenol (in the light green shadow). In plants, the OSCs enable a chair-chair-chair (CCC) conformation resulting in triterpenes as β-amyrin and friedelin (in the light green shadow).

Friedelin is a plant secondary metabolite from the class of triterpenes, molecules of 30-carbon skeleton derived from isoprenyl conjugation. Sterols have the same origin and are involved in primary needs of eukaryotic cells (from fungi to mammals), such as membrane stability and hormone production [1]. 2,3-Oxidosqualene is the common precursor of both sterol and triterpene molecules and it is folded in the active site of oxidosqualene cyclases (OSC) in two main conformations: chair-boat-chair or chair-chair-chair for sterol or triterpene synthesis, respectively. Then, it is protonated at the epoxide to initiate cyclization and carbocation rearrangements, ending with the formation of tetracyclic sterols or tetracyclic or pentacyclic triterpenes, depending on the cyclase product-specificity [2,3]. Friedelin is the pentacyclic triterpene with the maximum number of rearrangements and its aspartate residue in the synthase active site aids the production of a ketone group at C-3 instead of an alcohol (Figure 1), which is very common among pentacyclic synthases [4,5].

In fungi and mammals, the main sterol formed by OSC is lanosterol, which is oxidized to ergosterol or cholesterol, respectively [6]. Plants can form the tetracyclic cycloartenol and oxidize it to phytosterols such as stigmasterol and β-sitosterol [7]; they also oxidize the pentacyclic triterpenes into an array of molecules with the P450 enzyme family playing a significant part in those biosynthetic pathways [5]. Either way, the OSC substrate in eukaryotic cells is the same and the diverse final cyclized C-30 molecules can be functionalized into many other different compounds with different properties (Figure 1).

Friedelin accumulates in the leaves of *Maytenus ilicifolia* (Mart.) ex. Reissek (Celastraceae), which is native to South America [8]. *M. ilicifolia* is popularly used for stomach disorders due to the abundant presence of friedelin, an anti-inflammatory agent with gastroprotective properties [8,9,10]. Friedelin is also the precursor of the anticancer triterpene quinonemethides maytenin and pristimerin, biosynthesized by P450 oxidoreductases only in the root barks of Celastraceae species [11,12,13,14].

So far, the friedelin synthase genes from only two species have been functionally described: *Kalanchoe daigremontiana* [4] and *M. ilicifolia* [15]. There is a recent deposit in GenBank of a friedelin synthase sequence from *Populus davidiana* (GenBank accession number: ART66198.1), but no functional characterization is associated with this entry. This paper reports our recent observation of the presence of different polymorphisms of the friedelin synthase gene from *M. ilicifolia*, whose coding sequences were cloned and expressed in yeast and resulted in the slightly different production of friedelin.

## 2. Results and Discussion

### 2.1. There Are Four Coding Sequences for Friedelin Synthase in M. ilicifolia

Friedelin synthase from the leaves of *M. ilicifolia* (*Mi*FRS, GenBank accession number KX147270.1) was cloned in our previous work along with one cycloartenol synthase (GenBank accession number KX147271.1). Considering that plants have in average ten genes encoding triterpene synthases [16], we expected to clone more OSC coding sequences from *M. ilicifolia*. Therefore, we decided to re-analyze the cDNA synthesized from mRNA from the leaves using the primers for amplification of the full-length friedelin synthase (*Mi*FRS) and screen for other isoforms in its coding sequence (cds).

Three more coding sequences of the same size as *Mi*FRS were obtained and named as *Mi*FRS with numbers 2, 3 and 4. Multiple global alignment of the nucleotide sequences showed high identity to the previous cds of friedelin synthase first cloned from that species, as shown in Table 1.

After identification of the open reading frames, it was possible to compare the identity among the four translated protein sequences which also indicated an identity higher than 99%. The amino acid sequence of *Mi*FRS3 is 100% identical to *Mi*FRS4 although nucleotide sequences showed polymorphic positions. Detailed position of the single nucleotide polymorphisms (SNPs) among the sequences are presented in Table S1. Due to the high identity among the *Mi*FRS proteins, other features as molecular weight and pI showed little difference (Table 1).

Using the Identical Protein Groups tool from the National Center for Biotechnology Information (NCBI), we searched by the name of pentacyclic triterpene synthases as β-amyrin and lupeol synthases and observed, respectively, 45 and 20 different groups constituted of three to 25 identical deposits of the protein in each, according to a reference sequence. With further detailed search, we observed that 27 entries showed proteins with 99% of identity among several species like *Glycyrrhiza glabra*, *Solanum lycopersicum*, *Glycine max*, *Vitis vinifera*, *Nicotiana tabacum*, *Panax ginseng*, *Medicago truncatula*, *Avena strigose* and *Lotus japonicus*.

Considering the species *G. glabra*, there are seven accession numbers for β-amyrin synthase and two proteins shared 99.48% of identity. From this same species, reference β-amyrin synthase (UniProtKB/Swiss-Prot Q9MB42.1) showed 99% identity with 99% coverage with β-amyrin synthases identified in *Glycyrrhiza inflata* and *Glycyrrhiza uralensis*.

Those findings in the literature indicated the high identity of the same functional OSC among deposited sequences either from the same species or different ones. In the same way, we could also observe four different sequences with identity higher than 99% for friedelin synthase in *M. ilicifolia*.

**Figure 2:**
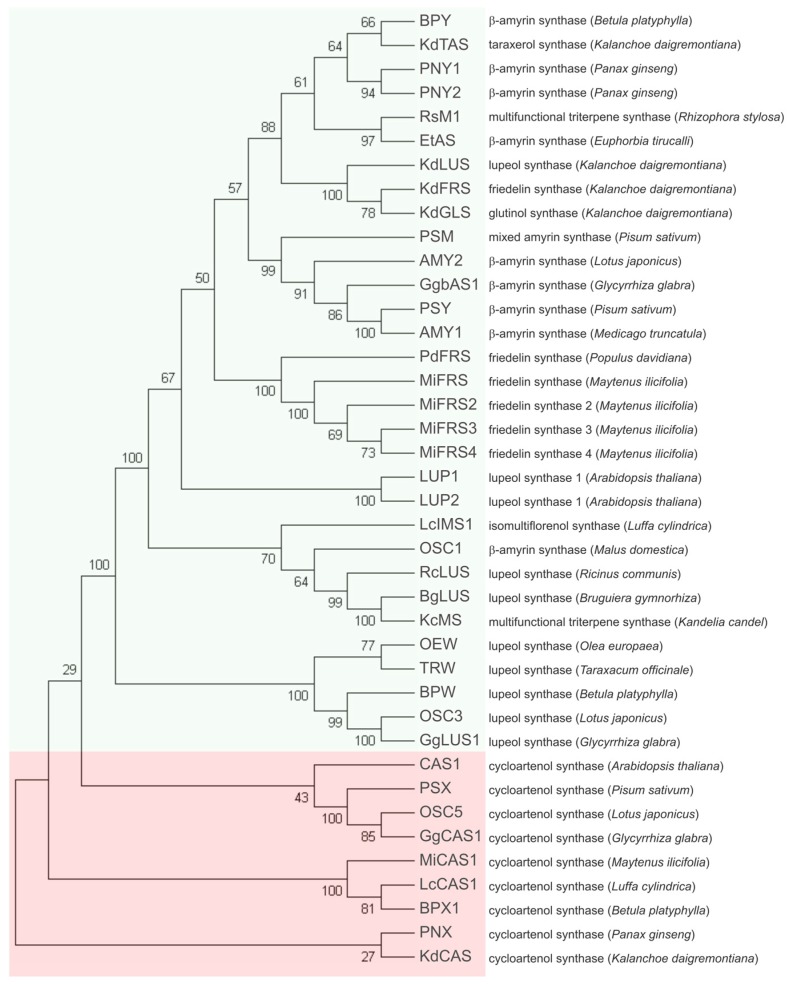
Phylogenetic analysis of *Mi*FRS among deposited protein sequences of other OSCs. The global multiple alignment among protein sequences of OSCs from different plants (accession data shown in Table S2) was done using ClustalW tool and phylogenetic tree was built in MEGA7 software. The percentage of replicate trees in which the associated taxa clustered together in the bootstrap test (1000 replicates) are shown next to the branches. Green box shows the clusterization of pentacyclic triterpene synthases, separated from the group of cycloartenol synthases, whose cluster is marked in the light red box. The group of friedelin synthases from *M. ilicifolia* is highlighted in green. Notice that *Pd*FRS and *Mi*FRS probably have a common ancestor.

Phylogenetic analysis by neighbor-joining clustered the sequences of *Mi*FRS in the branch with other pentacyclic triterpene synthases, separated from the cycloartenol synthases branches, as shown in Figure 2. The close clustering of the four *Mi*FRS confirmed their proximity identity when compared with other OSC from the databases (Table S2) and *Mi*FRS2 to 4 are derived from the first cloned enzyme. It is likely that these four sequences have a common ancestor with friedelin synthase from *Populus davidiana* (*Pd*FRS), since they shared the same branch in the evolutionary analysis. Friedelin synthase from *Kalanchoe daigremontiana* (*Kd*FRS) and its glutinol and lupeol synthases clustered close to each other but did not cluster with other friedelin synthases. The separated clusterization among friedelin synthases might be due to the divergence of OSCs in one species from an ancestral OSC gene, commonly resulting in OSCs of the same species being more related than OSCs with the same function from different species [16]. 

Using Blastp tool all *Mi*FRS proteins showed identity of 68–72% with high coverage (~98%) with β-amyrin synthases from different species and with *Pd*FRS (73%) but 66% with *Kd*FRS. As expected, cycloartenol synthases clustered together in a clade separated from the pentacyclic triterpene synthases.

### 2.2. MiFRS Gene Characterization and SNPs

**Figure 3:**
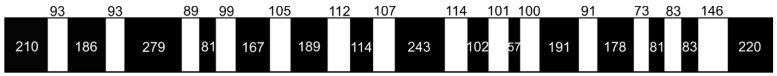
Exon-intron structure of *Mi*FRS gene. Prediction was obtained from Splign Alignment Tool (https://www.ncbi.nlm.nih.gov/Web/Newsltr/V14N2/splign.html) from NCBI. Exons are represented by black boxes. Numbers represent exon and intron lengths in base pairs.

We also cloned the genomic *locus* sequence of the friedelin synthase gene from DNA extracted from the leaves of *M. ilicifolia*. Analysis of the genomic sequence showed a fragment of about 4 kb presenting 14 introns and 15 exons. Figure 3 shows length of exon and intron sequences based on in silico prediction by pairwise alignment with *Mi*FRS cds. Most of the intron flanking sequences obeyed the splicing consensuses [17] as shown in Table S3. Similar genomic feature was shown by other oxidosqualene cyclases from plants as for the lupeol synthases of *Arabidopsis thaliana*, with 17 exons and 16 introns [18], β-amyrin synthase from *A. strigosa* [19], with 18 exons and 10 full-length genes from *Oryza sativa*, with 18 exons and one with 10 exons [20].

Observing the gene sequencing electropherograms (Figure S1), it was possible to detect the variants presented by the cDNAs calculating the peak height ratio. When a diploid organism presents heterozygosity for one gene, two peaks of same height are shown in the chromatogram, but polyploidy or diverse copy number of a gene can result in electropherograms with peaks with different heights for the variant nucleotide [21]. Unfortunately, the ploidy of *Maytenus* spp or the copy number of friedelin synthase gene, which could confirm that the different ratios of the found nucleotide variants represent the difference of the gene duplication, are still unknown. However, it is already known that the diverse OSCs come from divergent duplication events with neofunctionalization [16]. That information sheds light on the fact that variants of friedelin synthase encountered in *M. ilicifolia* might be related to duplication gene events in the species and different copy number of the variant sequences.

**Figure 4:**
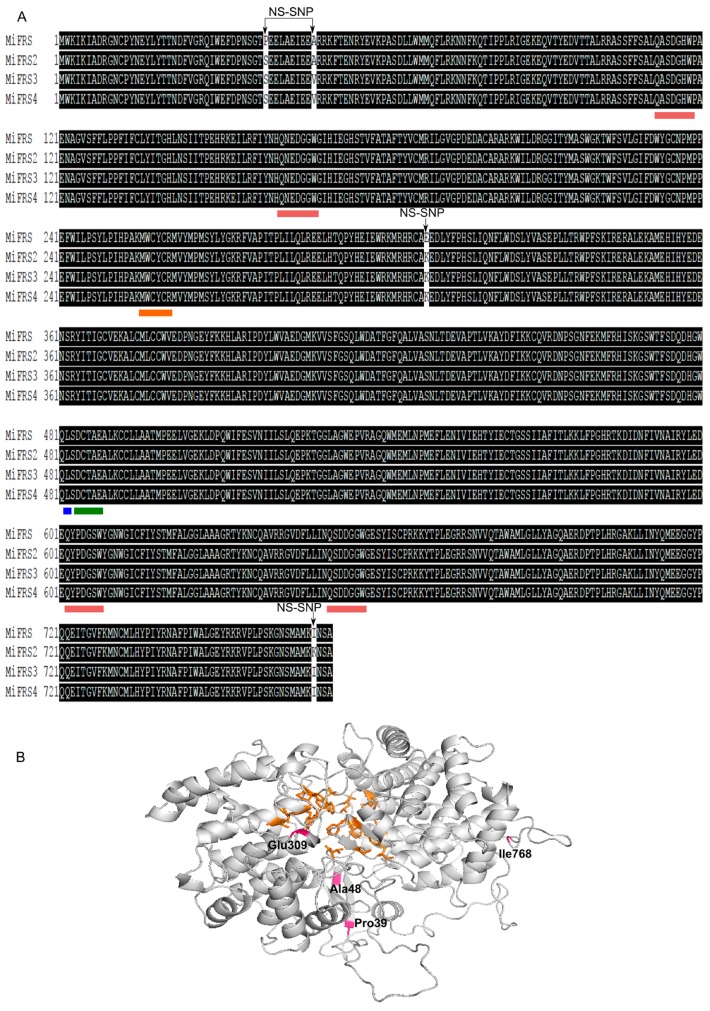
Amino acid sequence and structure of *Mi*FRS highlighting non-synonymous single nucleotide polymorphisms (NS-SNPS). (**A**) Global alignment of the four sequences cloned for *Mi*FRS with NS-SNPs shown. Boxes are delimiting the four structure QW-motifs (in pink) and the residues involved in the active site (orange, blue and green); (**B**) Homology model obtained for *Mi*FRS [15], illustrating that substituted residues (pink) do not interact with residues in the active site (orange).

Sequencing of the general coding DNA for *Mi*FRS sequences allowed the localization of 8 SNPs in exon 1, 5 and 15 (Figure 3). From those, four single nucleotide variants were non-synonymous when we observed the multiple global alignment of amino acid predicted sequences of the four proteins (Figure 4A) and always correlated to a substitution for the same group nucleotide (G/A or C/T) in position 1 or 2 of the codon (Table S1).

Non-synonymous single nucleotide polymorphisms (NS-SNPs) were observed in *G. uralensis* population, allocated in the first exon coding the enzyme [22] and four genotypes were confirmed, in which three of them are correlated with the high content of glycyrrhizic acid [23]. Therefore, analysis of the variants observed in the present study in different *Maytenus* individuals can validate these variants as SNPs that can be used as markers of the species with high production of friedelin.

### 2.3. MiFRS NS-SNPs and Enzyme Structure 

From the 9 SNPs observed among the four *Mi*FRS, four NS-SNPs were observed. In *Mi*FRS2, the Pro39 residue showed a missense mutation to a smaller polar serine residue, and both Glu309 and Ile768 were changed to a large and basic lysine residue. *Mi*FRS3 and 4 maintained the Ser39 and Lys309 but also presented the substitution of the aliphatic very small Ala48 to a medium sized aliphatic valine residue (Figure 4A).

All four proteins conserved the squalene cyclase domain subgroup 1 (SQCY_1) from residues 101 to 751 and isoprene-C2-like reductase (ISOPREN_C2) from residues 100 to 751. Missense mutations did not alter characteristic motifs already reported for the OSC family and friedelin synthase (Figure 4A): the four structural QW motifs (aromatic rich motifs of seven residues from Gln to Trp); the cavity site involving Met-Trp-Cys-Tyr-Cys-Arg motif; the Asp-Cys-Thr-Ala-Glu (DCTAE) catalytic site and the Leu482. All of them were reported to be important for product specificity [15].

When analyzing the missense residues in the isoforms of *Mi*FRS, it was interesting to note that the non-polar Pro39 is a well-conserved residue among other OSC as seen by global multiple alignment among them. However, the new isoforms presented a polar Ser39 residue substitution. On the other hand, Glu309 in *Mi*FRS is not a common residue among OSCs; besides *Mi*FRS, it is only observed in the new friedelin synthase identified from *P. davidiana*, whereas the correspondent Lys309 of the variants is found in most of OSCs. Both residues Ala48 and Val48 have similar chemical properties and are found in most of the OSCs at this position, while Ile768 is a residue just common in friedelin synthases from *M. ilicifolia* (Figure S2). Anyway, the residues of the missense mutations were positioned outside of the enzyme active site, at the end of β-sheets (Pro39 and Ala48) or in loops (Glu309 and Ile768) (Figure 4B). Considering the friedelin production specificity, it is still noteworthy to mention the maintenance of a leucine residue close to the catalytic aspartate only in the friedelin synthases (all *Mi*FRS isoforms, *Kd*FRS and the multifunctional *Kd*GLS and *Pd*FRS) in opposition to other pentacyclic triterpene synthases which have a valine residue in the correspondent position [4,15] (Figure S3).

### 2.4. Functional Characterization of the Variant Sequences

For functional characterization, the three variant sequences of *Mi*FRS were cloned into a yeast expression plasmid inducible by the strong promoter of *GAL1* (pYES2) and transformed in the *Saccharomyces cerevisiae* strain VZL1303, which presents a decreased abundance of *ERG7* mRNA, decreasing the competition of its encoding OSC protein (lanosterol synthase) for the same substrate 2,3-oxidosqualene. Since *Mi*FRS3 and *Mi*FRS4 presented the same amino acid composition, friedelin production was evaluated for *MiFRS*1, *Mi*FRS2 and *Mi*FRS4 and compared to friedelin production by *Kd*FRS enzyme.

**Figure 5:**
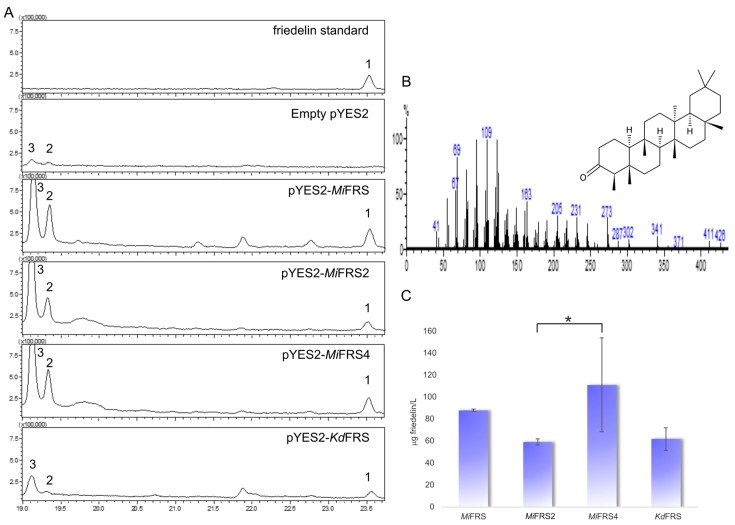
Friedelin production by the *Mi*FRS sequences and *Kd*FRS. (**A**) Total ion chromatograms (TIC) for friedelin (1) standard and in the different samples from yeast extract, except in the yeast extract from empty vector sample. Peaks 2 and 3 correspond to the accumulation of compounds from the ergosterol pathway, namely 4,4-dimethylzymosterol and lanosterol (mass spectra in Figure S4); (**B**) One of the mass spectra with the fragmentation patterns for friedelin, used to confirm its production in the yeast extracts. (**C**) Quantified production of friedelin in the different samples. Values are denoted in µg of friedelin by liter of cultivated inocula of strain VZL1303, as the average of three different extracts with standard deviation bars. Asterisk (*) represent significative statistical difference only between *Mi*FRS2 and *Mi*FRS4 the samples according to Tukey’s analysis (*p* ≤ 0.05). However, production of friedelin by *Mi*FRS and *Kd*FRS are not significantly different between each one or compared to *Mi*FRS2 and *Mi*FRS4.

After cultivating the strains in medium with galactose for induction of expression of *Mi*FRS variants, a non-polar extract of the cells was generated and gas-chromatography associated to mass spectrometry was used to identify the isoprenoids in the extract. All sequences produced friedelin as sole heterologous triterpene, that was not detected in the strain carrying an empty vector (Figure 5A) and showed the same retention time and mass spectra (Figure 5B) as the standard. Therefore, all variants of friedelin synthase cloned from the leaves of *M. ilicifolia* confirmed to be functional and specific for friedelin production.

Although the missense mutations did not change friedelin specificity, the productivity of each new *Mi*FRS sequence in yeast was explored in order to select the best enzyme sequence for improving the heterologous production. As mentioned before, friedelin is an anti-inflammatory pentacyclic triterpene that is also the precursor of important anticancer triterpenic quinonemethides produced in low amount in the barks of the roots of *M. ilicifolia* [14]. Therefore, improvements in the yeast production of these metabolites can promote their future therapeutic use. Maintenance of their production levels constantly and in suitable amount without environment changes and exploitation of plant species are some of the microbial production advantages as well as rapidity and economy [24].

Quantification of friedelin in the four samples showed a heterologous production higher than 50 µg of friedelin per liter of inoculum. When comparing the production among the four friedelin synthase sequences, *Mi*FRS2 produces statistically significant less friedelin than *Mi*FRS4 (Figure 5C), but *Mi*FRS1 and *Kd*FRS had not statistically different production among the other enzymes (considering *p* < 0.05). However, the *Kd*FRS sequence used in the present study was bought with codon optimization for *S. cerevisiae* whereas all *Mi*FRS codon sequences were the same as in plant and not optimal for the preferential codons in yeast. In that sense, this data suggests that *Mi*FRS had a promising production in the yeast. Otherwise, it is interesting to observe accumulation of precursors of the ergosterol pathways, as 4,4-dimethylzymosterol and lanosterol (compounds in the peaks indicated as 2 and 3, respectively, in Figure 5A). To improve friedelin production, the accumulation of lanosterol should be avoided. Lanosterol is also a product of 2,3-oxidosqualene cyclization [25], thus consuming the friedelin synthase substrate. However, the gene encoding lanosterol synthase is essential and cannot be deleted without ergosterol addition [4], what raises the costs of the production. So, controlling of the synthase can be one of the first modifications in *S. cerevisiae*, as long as overexpressing genes of other enzymes related to the production of 2,3-oxidosqualene [26].

A similar report was done for two genotypes of β-amyrin synthase from *G. uralensis*. When expressed in *S. cerevisiae*, the isoform with one missense mutation from Gly to Asp (at nucleotide 94, G/A) and one synonymous (at nucleotide 254, C/T) showed β-amyrin production 10-fold higher [27]. In our experiment, we did not see significative improvement in friedelin production according to statistical analysis, so *Mi*FRS or *Mi*FRS4 were the best friedelin producers in *S. cerevisiae* and should be used for synthetic biology further experiments.

## 3. Materials and Methods

### 3.1. Plant Material

Leaves of *M. ilicifolia* were collected at the School of Pharmaceutical Sciences, São Paulo State University, Araraquara, Brazil and identified by Prof. Dr. Julio A. Lombardi. A voucher specimen was deposited under number HRCB 68663 at the “Herbário Rioclarense” in the São Paulo State University (UNESP), Rio Claro, SP, Brazil.

### 3.2. OSC Cloning

OSC cloning was based on previous work [15]. Briefly, harvested leaves were immediately frozen in liquid nitrogen and total RNA was extracted using the guanidine hydrochloride buffer (buffer RLC) and the RNeasy Plant Mini Kit (Qiagen, Hilden, Germany). Synthesis of cDNA was performed with High-Capacity cDNA Reverse Transcription kit (Applied Biosystems, Foster City, CA, USA). The synthetized cDNA was used for full-length ORF amplification with specific primers (Table S4) and PCR was performed with Platinum *Pfx* DNA Polymerase (2.5 U/μL; Invitrogen, Carlsbad, CA, USA), according to the conditions of hot start at 94 °C for 2 min, 30 cycles of 94 °C for 30 s, 55 °C for 45 s and 68 °C for 3 min, and a final extension at 68 °C for 10 min. Resulting ~2.3 kb PCR products were cloned into pTZ57R/T vector (Fermentas, Waltham, MA, USA) and were heat shock transformed into competent *Escherichia coli* DH10B strain. Recombinant plasmids were purified from bacterial cells with QIAprep spin miniprep kit (Qiagen) and sequenced in a Genetic Analyzer 3130 (Applied Biosystems) after PCR with primers in Table S3. Sequences were aligned against *Mi*FRS sequence previously identified using Clustal Omega (https://www.ebi.ac.uk/ Tools/msa/clustalo/). GenBank accession number, of the cloned sequences are: MG677552, MG677553 and MG677554, for *Mi*FRS2, *Mi*FRS3 and *Mi*FRS4, respectively.

### 3.3. Phylogenetic Analysis

OSC sequences from different plants were recovered from GenBank, NCBI reference sequence and UniProtKB (Table S2) and were aligned with the four sequences cloned in this study using ClustalO (https://www.ebi.ac.uk/Tools/msa/clustalo/). The evolutionary tree was constructed using the Neighbor-joining method with default parameters based on 1000 bootstrap replicates in Mega 7 software (http://www.megasoftware.net/home) [27,28].

### 3.4. MiFRS Gene Sequencing

Frozen leaves (100 mg) of *M. ilicifolia* were also used for genomic DNA extraction. Extraction method comprised cell lysis by CTAB buffer with β-mercaptoethanol at 65 °C for 60 min, harvesting of DNA from aqueous phase after cell debris removal with chloroform:isoamyl alcohol (24:1, *v*/*v*), DNA purification by precipitation with high salt solution, ethanol wash and RNase and proteinase K treatment [29]. Primers used for ORF cloning were also used for *Mi*FRS gene amplification from the beginning of the first exon to the end of the last using High Fidelity PCR Enzyme Mix (5 U/µL; Thermo Scientific, Waltham, MA, USA) and conditions: hot start at 94 °C for 3 min, 30 cycles of 94 °C for 30 s, 55 °C for 45 s and 68 °C for 6 min, and a final extension at 68 °C for 10 min. Product PCR was purified and submitted to sequencing PCR with primers in Table S3. Pairwise alignment of the gDNA sequence and ORF (https://www.ncbi.nlm.nih.gov/sutils/splign/splign.cgi?textpage=online&level=form) allowed us to propose the length and position of introns and exons. Exon-intron flanking interfaces were predicted at http://www.cbs.dtu.dk/services/NetGene2/. GenBank accession number for the gene sequence is MG677133. SNPs were calculated by the ratio between peaks of different fluorophores based on electropherograms from gene sequencing using QSVanalyser (http://dna.leeds.ac.uk/qsv/) [21].

### 3.5. In Silico Analysis

Entry diversity and identity among β-amyrin synthases were verified by IPG (https://www.ncbi.nlm.nih.gov/ipg). ORF and amino acid sequence of each new isoform were determined by ORF Finder software (http://www.bioinformatics.org/sms2/orf_find.html). Global multiple sequence analysis was performed with Clustal Omega (https://www.ebi.ac.uk/Tools/msa/clustalo/) and Blastp tool (https://blast.ncbi.nlm.nih.gov/Blast.cgi) was used to compare deduced amino acids to deposited protein sequences. Conserved OSC domains were identified by NCBI Conserved Domain Search tool (http://www.ncbi.nlm.nih.gov/Structure/bwrpsb/bwrpsb.cgi). Molecular weight and pI were predicted at http://web.expasy.org/compute_pi/.

Phylogenetic analysis was performed by ClustalW alignment with 40 OSC plant sequences recovery from NCBI database and originated from this study. Phylogenetic tree was constructed by Neighbor-Joining method with MEGA7 default parameters and bootstrap from 1000 replicates.

### 3.6. Functional Analysis

The sequences of *Mi*FRS were subcloned to pYES2 yeast expression vector (Invitrogen) using *Bam*HI and *Eco*RI (Thermo Scientific). Plasmids were transformed by heat shock using lithium acetate and single-stranded DNA into strain VZL1303 (genotype *MAT*a, *Δura3*, *Δhis3*, *Δleu2*, *ERG7-KanMX*) and transformants were selected on SC medium (0.67% yeast nitrogen base, 2% glucose) without uracil (SC-ura). Yeast codon optimized *Kd*FRS (Epoch Life Science, Missouri, TX, USA) was also cloned into pYES2 and transformed into the same strain. Cells were cultivated in SC-ura and heterologous expression was induced by adding 2% galactose, for 10 h at 30 °C with shaking. Friedelin production was accomplished by incubating cells in 0.1 M potassium phosphate, pH 7.0 with 3% glucose for 24 h at the same temperature [4]. Then, cells were collected, dried and about 30 mg were extracted with chloroform:methanol (2:1, *v*/*v*) [30] in an ultrasonic bath (2840D, Odontobrás, Ribeirão Preto, SP, Brazil) for 10 min. Organic phase was collected after addition of 0.73% NaCl and centrifugation. Extract was dried and resuspended in 200 µL acetonitrile.

### 3.7. Chemical Analysis and Friedelin Quantification

Triterpene extract was submitted to gas chromatography associated to mass spectrometry (QP2020C W/O RP230V, Shimadzu, Kyoto, Japan) using a HP-5 column (30 m × 0.25 mm × 0.25 μm; Agilent Technologies, Santa Clara, CA, USA). Analysis was performed with inlet temperature of 270 °C, heating gradient from 200 to 290 °C (10 °C/min), trap temperature of 200 °C, interface temperature of 290 °C for 18 min, injection volume of 1 µL, split ratio of 1:10, flow gas of 1.0 mL/min, ionization of 70 eV and detection interval of 35 to 600 *m*/*z*. Structures from detected peaks were searched against the National Institute of Standards and Technology (NIST, Gaithersburg, MD, USA) library. Cholesterol was spiked in as internal standard control at 40 µg/mL before triterpene extraction process. An analytical curve of friedelin standard (Sigma-Aldrich, St. Louis, MO, USA) was constructed. Quantification analysis was done in triplicate and statistical significance was calculated by ANOVA and Tukey’s test (*p*-value < 0.05).

## 4. Conclusions

*M. ilicifolia* is a plant species with wide medicinal use due to its anti-inflammatory properties and it is also a source of anticancer quinonemethide triterpenes. Despite its therapeutic importance, no in depth studies of its genomic and transcriptomic analysis are described for this species [8]. In that sense, our group undertook a study of isoforms of friedelin synthase that could result in higher yields of the triterpene friedelin. However, in yeast, the four isoforms presented a similar production of friedelin and further improvement may be achieved by metabolic engineering of *S. cerevisiae*. Nonetheless, this work exploited the polymorphisms presented by genes of friedelin synthase in *M. ilicifolia* and population genotype analysis might validate those SNPs for allelic characterization of high friedelin producer specimens.

## Figures and Tables

**Table 1:** Identity and features among the four cds and ORFs of *Mi*FRS.

ORF Name	*Mi*FRS (KX147270.1)	*Mi*FRS2 (MG677552)	*Mi*FRS3 (MG677553)	*Mi*FRS4 (MG677554)
Nucleotide identity (%)	99.63 to *Mi*FRS2 and *Mi*FRS3; 99.67 to *Mi*FRS4	99.75 to *Mi*FRS3 and 99.79 to *Mi*FRS4	99.80 to *Mi*FRS4	-
ORF length (base pairs)	2316	2316	2316	2316
Amino acid length	771	771	771	771
Predicted MW (kDa)	89.16	89.16	89.17	89.17
Predicted pI	6.04	6.24	6.17	6.17
Protein identity (%)	99.61 to the other three	99.74 to *Mi*FRS3 and *Mi*FRS4	100 to *Mi*FRS4	-

ORF = open reading frame; MW = molecular weight; pI = isoelectric point; kDa = kilodaltons.

## Supplementary Materials

The following are available online.
