# Supplementary material for: Friedelin in Maytenus ilicifolia Is Produced by Friedelin Synthase Isoforms
Source: Molecules. 2018 Mar 20;23(3):700. doi: 10.3390/molecules23030700 (PMC6017009; doi:10.3390/molecules23030700)
Supplement: Supplementary file 1 [file molecules-23-00700-s001.pdf]

molecules

Friedelin in *Maytenus ilicifolia* Is Produced by Friedelin Synthase Isoforms

Thaís B. Alves [1], Tatiana M. Souza-Moreira [2], Sandro R. Valentini [2], Cleslei F. Zanelli [2,*] and Maysa Furlan [1]

[1] Instituto de Química, Univ. Estadual Paulista-UNESP, Rua Prof. Francisco Degni, 55, Quitandinha, Araraquara, SP 14800-060, Brazil; thais.barboni@gmail.com (T.B.A.); mayfurlan@gmail.com (M.F.)
[2] Faculdade de Ciências Farmacêuticas, Univ. Estadual Paulista-UNESP, Rod. Araraquara-Jaú km 1, Araraquara, SP 14801-903, Brazil; souzatm@gmail.com (T.M.S.-M.); valentsr@fcfar.unesp.br (S.R.V.)
* Correspondence: zanellicf@fcfar.unesp.br; Tel.: +55-16-3301-6996

Table S1. Comparison of variant sequences among *Mi*FRS sequences.

Sequence	Region	Allele	Synonymous	Codon position	Protein residue	Amino acid position
***Mi*FRS**	Exon 1	24C	yes	3	Asp	8
		81G	yes	3	Arg	27
		102C	yes	3	Pro	34
		115C	non	1	Pro	39
		143C	non	2	Ala	48
		195C	yes	3	Leu	65
	Exon 5	1295G	non	1	Glu	309
	Exon 15	3703T	non	2	Ile	768
***Mi*FRS2**	Exon 1	24T	yes	3	Asp	8
		81G	yes	3	Arg	27
		102T	yes	3	Pro	34
		115T	non	1	Ser	39
		143C	non	2	Ala	48
		195A	yes	3	Leu	65
	Exon 5	1295A	non	1	Lys	309
	Exon 15	3703A	non	2	Lys	768
***Mi*FRS3**	Exon 1	24T	yes	3	Asp	8
		81A	yes	3	Arg	27
		102T	yes	3	Pro	34

		115T	non	1	Ser	39
		143T	non	2	Val	48
		195A	yes	3	Leu	65
	Exon 5	1295A	non	1	Lys	309
	Exon 15	3703T	non	2	Ile	768
***Mi*FRS4**	Exon 1	24T	yes	3	Asp	8
		81A	yes	3	Arg	27
		102T	yes	3	Pro	34
		115T	non	1	Ser	39
		143T	non	2	Val	48
		195A	yes	3	Leu	65
	Exon 5	1295A	non	1	Lys	309
	Exon 15	3703T	non	2	Ile	768

Table S2. The sequences used in the phylogenetic analysis with their GenBank accession number, species and function.

Identification	Accession number	Species	Function
LUP1	GenBank: AEE36187.1	*Arabidopsis thaliana*	lupeol synthase 1
LUP2	GenBank: AEE36185.1	*Arabidopsis thaliana*	lupeol synthase 2
CAS1	GenBank: AAC04931.1	*Arabidopsis thaliana*	cycloartenol synthase
BPY	GenBank: BAB83088.1	*Betula platyphylla*	β-amyrin synthase
BPW	GenBank: BAB83087.1	*Betula platyphylla*	lupeol synthase
*Bg*LUS	UniProtKB: 8CDT3.1	*Bruguiera gymnorhiza*	lupeol synthase
*Et*AS	GenBank: BAE43642.1	*Euphorbia tirucalli*	β-amyrin synthase
*Ggb*AS1	GenBank: BAA89815.1	*Glycyrrhiza glabra*	β-amyrin synthase
*Kd*FRS	GenBank: ADK35125.1	*Kalanchoe daigremontiana*	friedelin synthase
*Kd*LUS	GenBank: ADK35126.1	*Kalanchoe daigremontiana*	lupeol synthase
*Kd*GLS	GenBank: ADK35124.1	*Kalanchoe daigremontiana*	glutinol synthase
*Kd*TAS	GenBank: ADK35123.1	*Kalanchoe daigremontiana*	taraxerol synthase

*Kc*MS	GenBank: BAF35580.1	*Kandelia candel*	multifunctional triterpene synthase
AMY2	GenBank: AAO33580.1	*Lotus japonicus*	multifunctional β-amyrin synthase
OSC3	GenBank: BAE53430.1	*Lotus japonicus*	lupeol synthase
*Lc*IMS1	UniProtKB: Q948R6.1	*Luffa cylindrica*	isomultiflorenol synthase
*Gg*LUS1	UniProtKB: Q764T8.1	*Glycyrrhiza glabra*	lupeol synthase
OSC1	NCBI Reference Sequence: NP_001280946.1	*Malus domestica*	β-amyrin synthase
*Mi*FRS	GenBank: APG38073.1	*Maytenus ilicifolia*	friedelin synthase
*Mi*FRS2	Under submission	*Maytenus ilicifolia*	friedelin synthase 2
*Mi*FRS3	Under submission	*Maytenus ilicifolia*	friedelin synthase 3
*Mi*FRS4	Under submission	*Maytenus ilicifolia*	friedelin synthase 4
AMY1	GenBank: AAO33578.1	*Medicago truncatula*	β-amyrin synthase
OEW	GenBank: BAA86930.1	*Olea europaea*	lupeol synthase
PNY1	GenBank: BAA33461.1	*Panax ginseng*	β-amyrin synthase
PNY2	GenBank: BAA33722.1	*Panax ginseng*	β-amyrin synthase
PSM	GenBank: BAA97559.1	*Pisum sativum*	mixed-amyrin synthase
PSY	GenBank: BAA97558.1	*Pisum sativum*	β-amyrin synthase
*Pd*FRS	GenBank: ART66198.1	*Populus davidiana*	friedelin synthase
*Rs*M1	GenBank: BAF80441.1	*Rhizophora stylosa*	multifunctional triterpene synthase
*Rc*LUS	NCBI Reference Sequence: NP_001310684.1	*Ricinus communis*	lupeol synthase
TRW	GenBank: BAA86932.1	*Taraxacum officinale*	lupeol synthase
BPX1	GenBank: BAB83085.1	*Betula platyphylla*	cycloartenol synthase
*Gg*CAS1	GenBank: BAA76902.1	*Glycyrrhiza glabra*	cycloartenol synthase

Table S2. *Cont*.

*Kd*CAS	GenBank: ADK35127.1	*Kalanchoe daigremontiana*	cycloartenol synthase
OSC5	GenBank: BAE53431.1	*Lotus japonicus*	cycloartenol synthase
*Lc*CAS1	UniProtKB: Q9SLP9.1	*Luffa cylindrica*	cycloartenol synthase
*Mi*CAS1	GenBank: APG38074.1	*Maytenus ilicifolia*	cycloartenol synthase 1
PNX	GenBank: BAA33460.1	*Panax ginseng*	cycloartenol synthase
PSX	GenBank: BAA23533.1	*Pisum sativum*	cycloartenol synthase

Table S3. Predicted exon-intron flanking consensus interfaces in *Mi*FRS gene sequence.

**Donor splice sites, direct strand, 5′exon^3′intron**	**Acceptor splice sites, direct strand, 5′intron^3′exon**
GATGATGCAG^GTACTATATT	
TCCTCCATTT^GTGAGTATTT	AAAACTGCAG^ATCTTTTGCC
TTGGTTTTCG^GTGCGATAAA	
ATACATCCAG^GTAACTAAGA	ATCAATTCAG^CAAAAATGTG
ATGCGCAGAG^GCATGGATTC	TACCTTGCAG^GAGGATCTGT
CGTTGAAAAG^GTATCAAATA	TATCGAGCAG^GCGCTGTGTA
GAAAGTGGTG^GTAAGTAGAG	TCATGTGCAG^AGTTTTGGCA
AGCCTTAAAG^GTAGTGAATT	TATTTTACAG^TGCTGCCTGC
CCTCCAAGAG^GTAAAATTGA	GTCATTGCAG^CCTAAAACTG
ATGGATAGAG^GTAACTAATT	CCTTTTATAG^ATGCTCAACC
ATGGCTCGTG^GTACATTCTC	GTTTATCTAG^GTATGGGAAC
CCCAAGAAAG^GTGCATTTTT	GGTGCTACAG^AAATATACGC
TGCTGGCCAG^GTGAAAACTC	CCCAAAATAG^GCTGAGAGAG
CCCTCAGCAG^GTACTTTCTT	

Table S4. Primers used in this study.

**Primer**	**Sequence 5′ – 3′**	**Function**
VZO1381	CGC<u>GGATCC</u>ATCATGTGGAAGATAAAGATTGC	Forward for full-length ORF amplification. *Bam*HI cleavage site. Also used for gene amplification and sequencing.
VZO1382	CG<u>GAATTC</u>CGTGTAAGTGGACATATCATATGA	Reverse for full-length ORF amplification. *Eco*RI cleavage site. Also used for gene amplification and sequencing.
M13F	GTAAAACGACGGCCAGT	ORF sequencing
M13R	CAGTATCGACAAAGGAC	ORF sequencing
VZO1473	GGGACACAGCACAGTGTTT	ORF and gene sequencing
VZO1471	CTCACAGATGAAGTAGCTCCTACTC	ORF sequencing
VZO1472	CCGAGTGCAAACATTGTAC	ORF sequencing
VZO1134	GGAGGGCTAGCAGCAGCTGG	ORF and gene sequencing
VZO1950	GGCATGTAAACCATCC	Gene sequencing
VZO1951	GGGTTGCTGAGGATGG	Gene sequencing
VZO1952	GGAGTTTCTTGAGAATATTGTG	Gene sequencing
VZO2067	CTGCTTCCGACCTCCTTTGG	Gene sequencing

Endonuclease restriction sites are underlined.

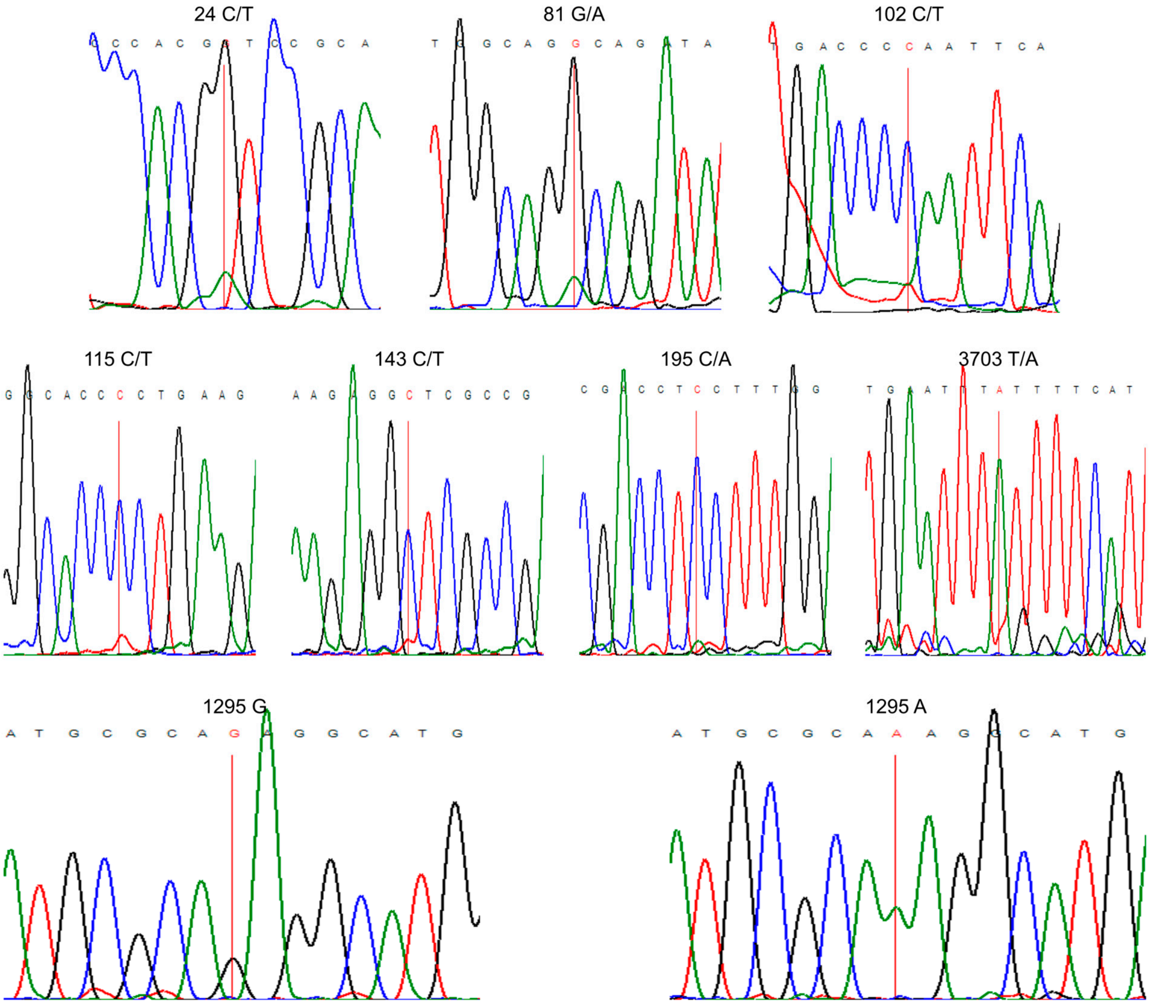

Figure S1. Electropherogram of SNPs detected in the gene of *Mi*FRS. Electropherograms correponding to nucleotide positions 24, 81, 102, 115, 143, 195, 3703 represent SNPs in exon 1 and 15. The position is indicated above each electropherogram, which were generated by sequencing of the gene PCR product. SNP in position 1295 correspond to SNP in exon 5, whose PCR product was cloned into plasmids and then sequenced individually. Therefore, it is possible to see one electropherogram for each polymorphism. Images were generated by the QSVAnalyser tool. Electropherogram for SNP 24 C/T and 3703 T/A were observed by using reverse primer for sequencing.

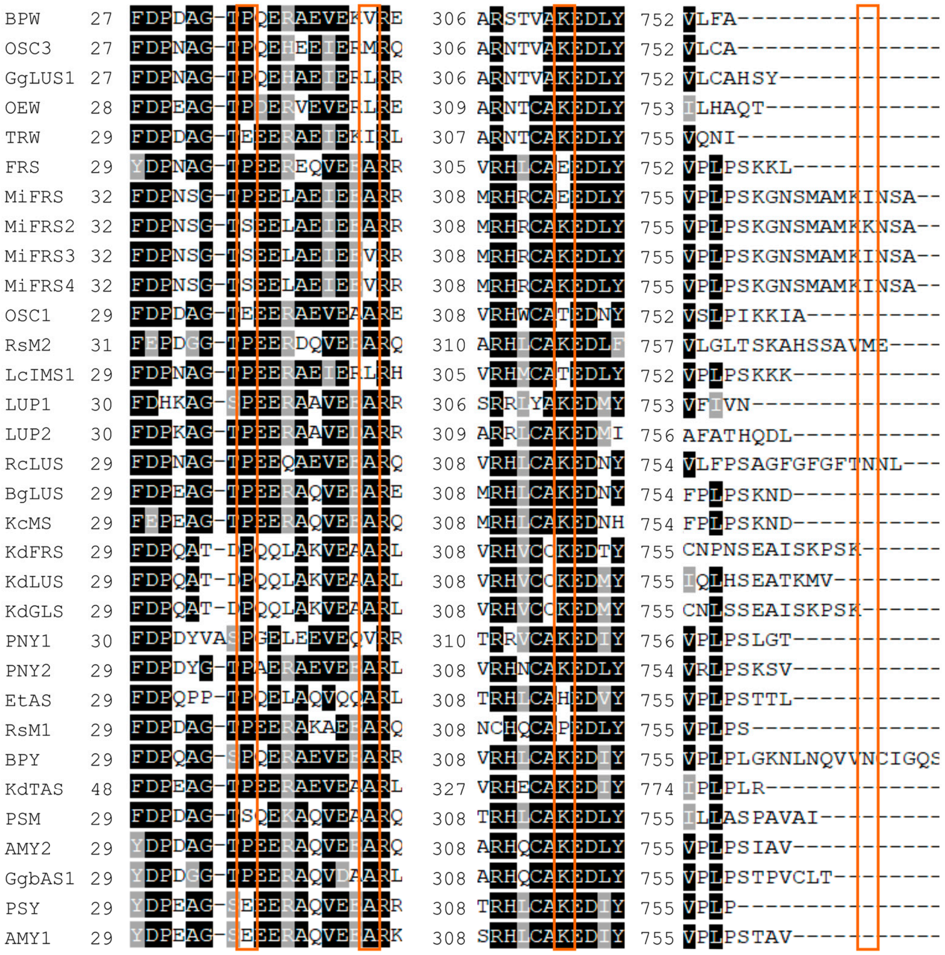

Figure S2. Multiple global alignment with diverse pentacyclic triterpene synthases. It is interesting to note the conservation of a Pro39 residue, an aliphatic residue at position 48, as Ala or Val and Lys309.

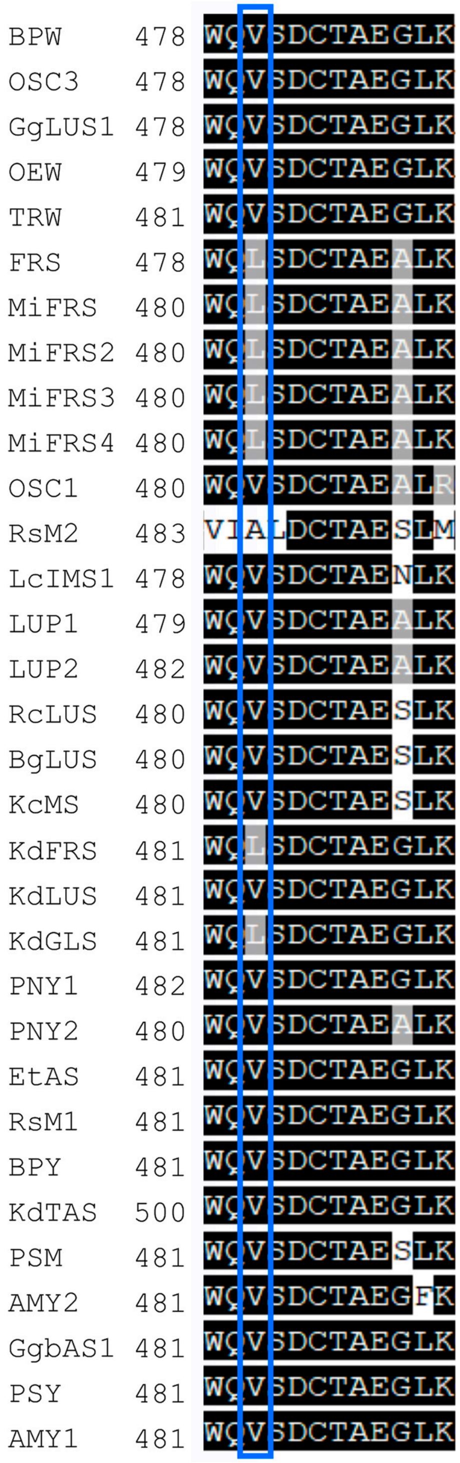

Figure S3. Leucine residue close to catalytic aspartate is conserved only in friedelin synthases. Multiple global alignment with diverse pentacyclic triterpene synthases shows the presence of a valine at the corresponding position in most of the enzymes, but not in the ones producing friedelin.

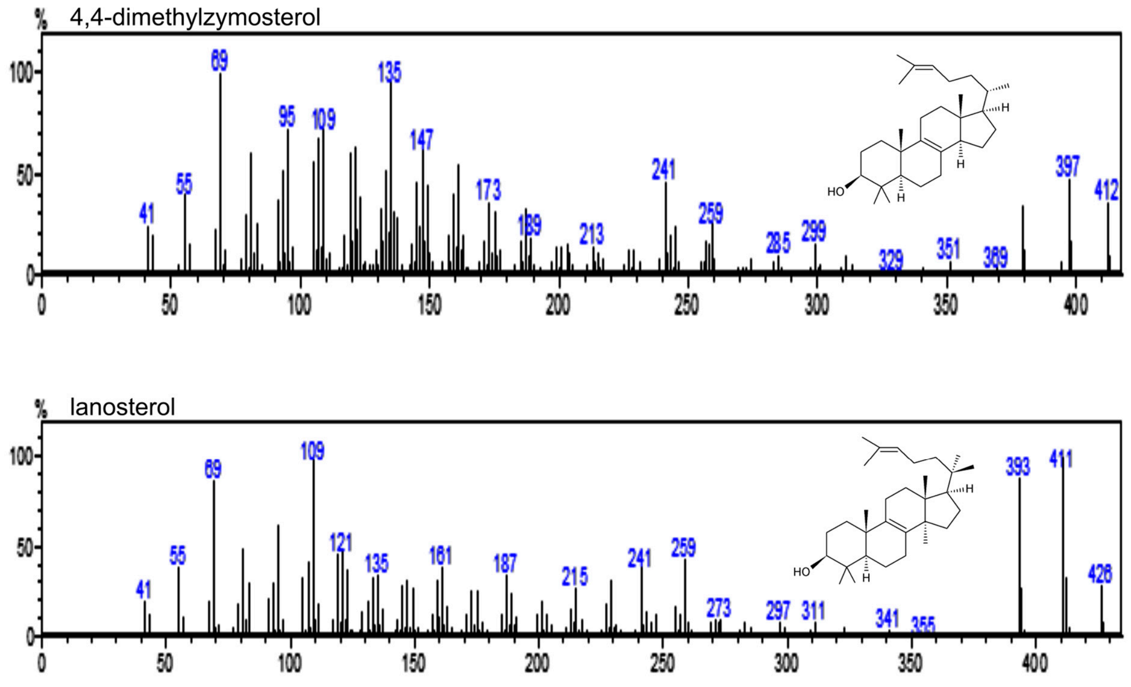

Figure S4. Mass spectra of 4,4-dimethylzymosterol and lanosterol.
